# Mitochondrial DNA and inflammatory proteins are higher in extracellular vesicles from frail individuals

**DOI:** 10.1186/s12979-023-00330-2

**Published:** 2023-01-30

**Authors:** Anjali M. Byappanahalli, Nicole Noren Hooten, Mya Vannoy, Nicolle A. Mode, Ngozi Ezike, Alan B. Zonderman, Michele K. Evans

**Affiliations:** 1 Laboratory of Epidemiology and Population Science, National Institute on Aging, National Institutes of Health, 251 Bayview Boulevard, Suite 100, Baltimore, MD 21224 USA; 2 Present address: Perelman School of Medicine, University of Pennsylvania,3400 Civic Center Boulevard Philadelphia, Philadelphia, PA 19104 USA

**Keywords:** Frail, Health disparities, EV, Exosome, Aging, Mitochondria, mtDNA, inflammation, Proteins, DAMP, Social determinants of health

## Abstract

**Background:**

Frailty, a clinical syndrome commencing at midlife, is a risk for morbidity and mortality. Little is known about the factors that contribute to the chronic inflammatory state associated with frailty. Extracellular vesicles (EVs) are small, membrane-bound vesicles that are released into the circulation and are mediators of intercellular communication. We examined whether mitochondrial DNA (mtDNA) and inflammatory proteins in EVs may act as damage-associated molecular pattern (DAMP) molecules in frailty.

**Results:**

To address whether EVs and their associated mtDNA and inflammatory protein cargo are altered with frailty, EVs were isolated from non-frail (*n* = 90) and frail (*n* = 87) middle-aged (45–55 years) participants from the Healthy Aging in Neighborhoods of Diversity across the Life Span (HANDLS) study. EV concentration was highest in frail White participants. EV mtDNA levels were significantly higher in frail individuals compared to non-frail individuals. The presence of six inflammatory proteins in EVs (FGF-21, HGF, IL-12B, PD-L1, PRDX3, and STAMBP) were significantly associated with frailty. EV inflammatory proteins were significantly altered by frailty status, race, sex, and poverty status. Notably, frail White participants had higher levels of EV-associated CD5, CD8A, CD244, CXCL1, CXCL6, CXCL11, LAP-TGF-beta-1 and MCP-4 compared to frail and non-frail African American participants. Frail White participants living below poverty had higher levels of EV-associated uPA. EV-associated CCL28 levels were highest in non-frail women and CXCL1 were highest in non-frail men. Men living below poverty had higher levels of CD5, CD8A, CXCL1, LAP-TGF-beta-1, and uPA. CXCL6 levels were significantly higher in individuals living above poverty. There was a significant correlation between EV mtDNA levels and the presence of inflammatory proteins.

**Conclusions:**

These data suggest that mtDNA within EVs may act as a DAMP molecule in frailty. Its association with chemokines and other inflammatory EV cargo proteins, may contribute to the frailty phenotype. In addition, the social determinant of health, poverty, influences the inflammatory cargo of EVs in midlife.

**Supplementary Information:**

The online version contains supplementary material available at 10.1186/s12979-023-00330-2.

## Background

Frailty is an age-associated clinical syndrome, characterized by a decline in physiological compensation and increased risk to stressors [1, 2]. Although frailty is usually examined in older populations, it commences at mid-life and is a risk for mortality at younger ages [3, 4]. Frailty prevalence increases with age but is also influenced by social determinants of health (SDOH). For example, frailty is prevalent for individuals living below poverty [3, 5]. Frailty is more prevalent in older (≥65 years, mean age = 73.6 years) African American adults than in White adults [6]. However, at specific ages in midlife (45–54 years), White adults have a higher prevalence of frailty than African American adults [3]. Frailty prevalence is also higher in women, compared to men; however, despite higher chronic disease burden, women have a longer lifespan compared to men [7–10]. Thus, the frailty phenotype is influenced by biological factors such as sex and age and SDOH such as race and poverty. Therefore, it is important to decipher underlying biological mechanisms that may drive frailty and identify biomarkers that may assist in early screening and diagnosis, especially to distinguish individuals at risk for premature mortality.

Frailty is a multisystem and complex condition that likely is the result of dysregulation of several pathophysiological processes. Chronic low grade sterile inflammation contributes to the frailty phenotype, through interfering with homeostatic tissue repair mechanisms leading to the accumulation of tissue damage [1, 2]. Frailty is also associated with higher levels of several proinflammatory cytokines, including IL-6 [11–13]. Furthermore, this chronic sterile inflammation which can begin at midlife is a risk for frailty later in life [14]. Sterile inflammation resulting from cellular stress and damage, ischemia, trauma, or environmental conditions causes the extrusion of cellular components and debris including damage associated molecular pattern (DAMP) molecules [15–17]. These DAMPs include nuclear and mitochondrial DNA (mtDNA), specific proteins, reactive oxygen species as well as other molecules [15–17]. DAMPs can then bind to specific pattern recognition receptors that can initiate a cascade of cellular signals that promote an inflammatory state. Therefore, tissue damage can spur a chronic feedback loop where DAMPs are released leading to a proinflammatory state and inflammation can then further inhibit maintenance and repair of tissue [15, 16]. However, we are only beginning to understand the relationship between DAMPs, inflammatory proteins, and frailty in humans and how this relationship may potentiate the frailty phenotype.

Circulating levels of specific DAMP molecules, circulating cell-free mitochondrial DNA (ccf-mtDNA), and circulating cell-free DNA (ccf-DNA), have been explored in the context of frailty. One study found that total ccf-DNA was associated with the inflammatory markers CRP and IL-6 and with frailty, while ccf-mtDNA copy number was correlated with frailty, but not CRP and IL-6 in individuals older than 90 years of age [18]. A study conducted by Ampo et al. found that ccf-mtDNA was significantly elevated in individuals who self-identified as pre-frail/frail having late-life depression compared to healthy, never-depressed individuals [19]. Therefore, we have yet to fully grasp the relationship between ccf-mtDNA, inflammation, and frailty.

Ccf-mtDNA in plasma can be encapsulated in extracellular vesicles (EVs) [20–22]. EVs are small, membrane bound vesicles that are important mediators of intracellular communication between cells [23, 24]. EVs can carry various cargo, including nucleic acids (DNA, various RNAs), lipids, and proteins [24–27]. There are several types of EVs that are released from cells (i.e. exosomes, microvesicles, and apoptotic bodies), but due to the difficulty in distinguishing the biogenesis pathway for these vesicles, the general term EV is used [28]. EVs can be isolated from biofluids making them attractive biomarkers for various conditions and diseases [23, 29–32].

Few studies have explored EVs in frailty. A small study of elderly adults (79–92 years) found no significant difference in serum EV concentration comparing frail (*n* = 5) and robust (*n* = 7) participants [33]. Another study explored microRNAs (miRNAs) in EVs as candidate biomarkers of frailty [34]. In this cohort (*n* = 14) of White individuals, eight miRNAs were enriched in frail individuals compared to either young individuals or robust elderly individuals: miR-10a-3p, miR-92a-3p, miR-185-3p, miR-194-5p, miR-326, miR-532-5p, miR-576-5p, and miR-760 [34]. Using immunoblotting as a semi-quantitative method for EV protein levels, levels of three mitochondrial specific proteins, adenosine triphosphate 5A (ATP5A; complex V), nicotinamide adenine dinucleotide reduced form (NADH): ubiquinone oxidoreductase subunit S3 (NDUFS3; complex I), and succinate dehydrogenase complex iron sulfur subunit B (SDHB; complex II), were lower in individuals with frailty and sarcopenia (*n* = 11) compared to individuals without sarcopenia and frailty (*n* = 10) [35]. These data indicate that there may be differences in mitochondrial components with frailty, but warrants follow up in a larger population using quantitative methods.

Thus far, there is limited information on EVs in the context of frailty, especially with relation to race, sex, and poverty status. This is important as there are disparities in frailty prevalence across all three demographic variables. Previously, our laboratory has shown that EVs can carry various DAMP molecules, inflammatory proteins, and ccf-mtDNA [20, 36, 37]. In this exploratory study, we examined whether EVs and their associated cargo including mtDNA and inflammatory proteins are altered with frailty in a middle-aged cohort of African American and White adults living above and below poverty.

## Results

### Plasma EV characteristics of frailty cohort

**Fig. 1:**
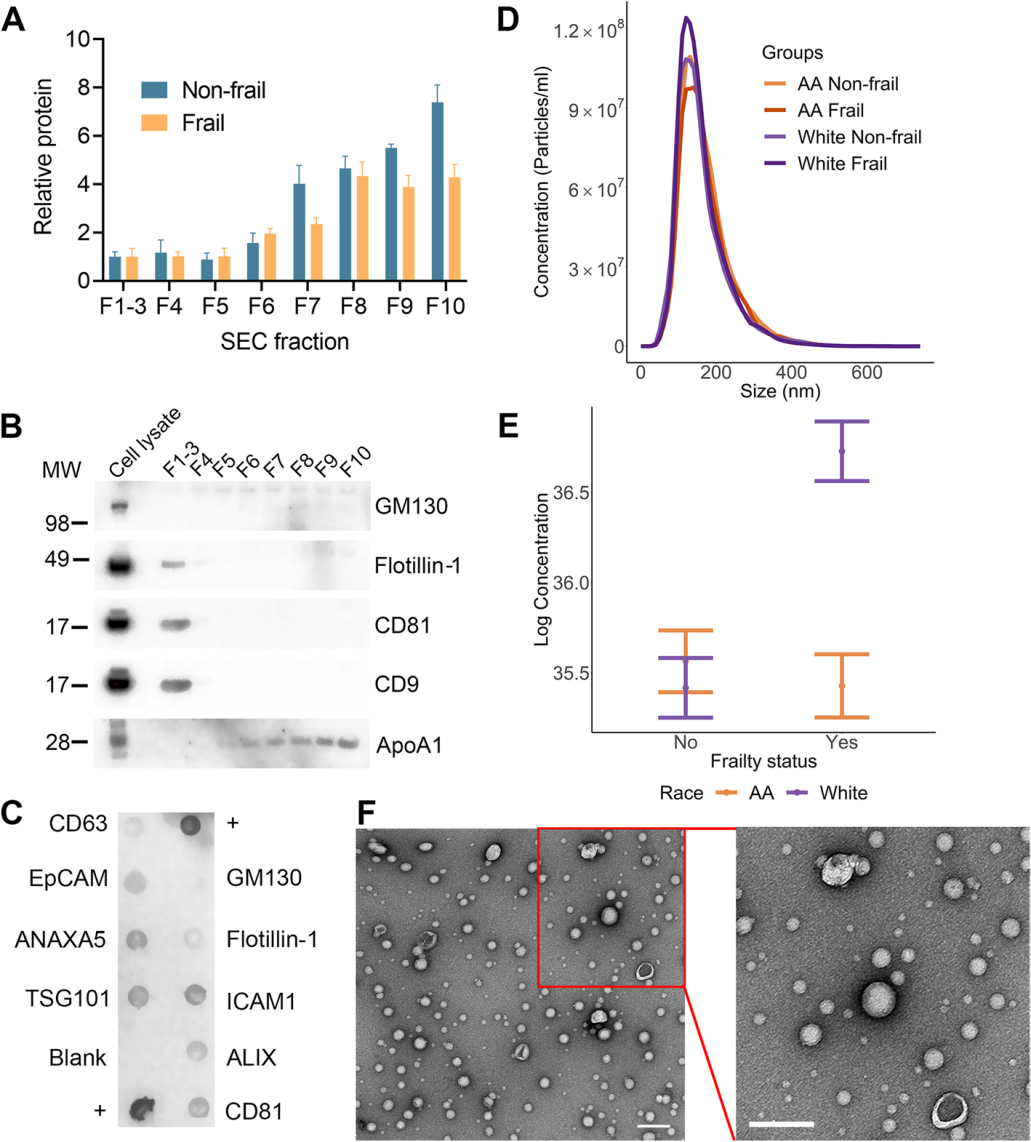
EV protein and concentration profile of the frailty cohort. **A **Plasma was separated using size exclusion chromatography (SEC) into 10 fractions that were lysed and relative protein concentration was determined. The extracellular vesicle (EV)-enriched fractions F1–3 were pooled. The histogram represents the relative mean protein concentration for fractions from non-frail (*n* = 3) and frail (*n* = 3) individuals + standard error of the mean. **B** SEC EV fractions and human umbilical vein endothelial cells were lysed and analyzed by SDS-PAGE and immunoblotted with antibodies against EV markers Flotillin-1, CD81, and CD9. GM130 and ApoA1 were used as purity markers. **C** Exo-Check™ Exosome Antibody Array was used to further validate EVs for common EV markers. Positive (+) control for the assay is indicated. **D** EV size and distribution were analyzed by nanoparticle tracking analysis, shown here by race and frailty status. The distribution was averaged for each group (African American non-frail *n* = 43, African American frail *n* = 41, White non-frail *n* = 47, White frail *n* = 46). **E** EV concentration values were log_2_ transformed. Linear regression was used to examine the relationship between EV concentration and frailty status, accounting for sex, race, and poverty status (*n* = 177). The plot shows the linear regression values ± standard error of the estimated values. **C)** EV morphology and size were visualized using electron microscopy. Region outlined in red was zoomed in for further visualization (scale bars = 200 nm). AA = African American; F = fraction

To assess whether EVs may be biomarkers of frailty and contribute to the frailty phenotype, we identified middle-aged individuals (45–55 years) in the HANDLS study who were frail (*n* = 87) and non-frail (*n* = 90) living above and below poverty, matched across sex and race (Table 1). EVs were isolated from plasma samples of these participants using size exclusion chromatography (SEC) with an Automatic Fraction Collector (AFC). The AFC facilitates a high-throughput approach for isolation of EVs from larger numbers of samples. Fractions 1–3 (F1–3) were pooled together, as they were the EV-enriched fractions. Consistent with other reports, the later fractions F4–10 were increasingly enriched in plasma proteins (Fig. 1A) [38, 39]. Isolated plasma EVs were validated according to Minimal Information for Studies of Extracellular Vesicles (MISEV) guidelines from the International Society of Extracellular Vesicles [40]. We assessed the presence of EV-associated markers by immunoblotting (Fig. 1B). The EV markers Flotillin-1, CD81, and CD9 were present in the EV-enriched fraction while the purity markers GM130 and ApoA1 were absent in the EV-enriched fraction. In addition, EV markers were also validated using an Exo-Check™ Exosome Antibody Array. The array confirmed the presence of other EV markers including ALIX, ANAXA5, CD81, EpCAM, ICAM1, and TSG101 (Fig. 1C). We further validated the size and concentration of the EVs using nanoparticle tracking analysis (NTA). The size distribution revealed a peak around 170 nm (Fig. 1D), which is consistent with EVs isolated from plasma. Through linear regression analysis, we found a significant interaction between frailty and race with EV concentration (*p* < 0.0001). Overall, EV concentration was highest in frail White participants, with significantly higher levels than non-frail White and frail African American participants (Fig. 1E). In Fig. 1D and E, we show the data for the distribution of EV concentration and size by frailty status and race, and interaction between frailty and race with EV concentration (Fig. 1D, E). Electron microscopy of the EV preparation showed a typical EV morphology with clear, round membranous vesicles and in some cases a cup-like shape, which can occur during the dehydration process for sample preparation in transmission electron microscopy (Fig. 1F) [41]. Collectively, these data indicate that plasma EVs isolated using SEC display characteristics that are attributed to EVs.

**Table 1:** Demographics for frailty sub-cohort of HANDLS study

Characteristic	Frail, *N* = 87	Non-frail, *N* = 90	***P***-value
Age	50.66 (2.62)	50.06 (2.84)	0.15
Men (%)	24 (28%)	25 (28%)	> 0.9
AA (%)	41 (47%)	43 (48%)	> 0.9
Below Poverty (%)	43 (49%)	38 (42%)	0.3

Age is reported as mean ± (SD), while n (%) are reported for sex, race, and poverty status. Student’s t-test was used to analyze differences among the groups for age. Pearson’s chi-squared tests were used to analyze differences for sex, race, and poverty status

*AA* African American

### EV-associated mtDNA levels and frailty status

Ccf-mtDNA can act as a DAMP molecule and can be present in plasma EVs [17, 20–22]. Here we hypothesized that EVs from frail individuals may contain higher levels of ccf-mtDNA, which may contribute to the chronic inflammatory state observed in frailty. To test this idea, we examined EV mtDNA levels in our cohort using an experimental pipeline that we previously reported [20] which was optimized here for utilization of SEC-isolated EVs. Details of this workflow can be visualized in Supplementary Fig. 1. First, plasma EVs isolated using SEC were DNase treated to remove any DNA outside of the EVs. Then, DNA was isolated and levels of EV mtDNA were measured by quantitative real-time PCR (qPCR) using four primer sets that target different regions of the mitochondrial genome (Supplementary Table 1; Supplementary Fig. 1). The primers were designed to hybridize against regions spanning between 16S rRNA (*MT-RNR2*) and tRNA-Ile1 (*MT-TL1*) genes (Mito_3164), the NADH dehydrogenase 2 (*MT-ND2*) gene region (Mito_4625), Cytochrome c oxidase subunit 2 (COX2) gene region (Mito_7878), and the ATP8 (*MT-ATP8*) gene region (Mito_8446) (Supplementary Fig. 1).

We examined the DNA isolated from plasma EVs from our frailty cohort. The relationship between EV mtDNA levels amplified with the four primer sets was analyzed using Pearson correlation. All four mtDNA amplicons positively correlated with each other, and these correlations were significant (Supplementary Fig. 2). Therefore, all four regions of the mitochondrial genome were present in the EVs and each of the different primer sets are highly correlated with each other.

**Fig. 2:**
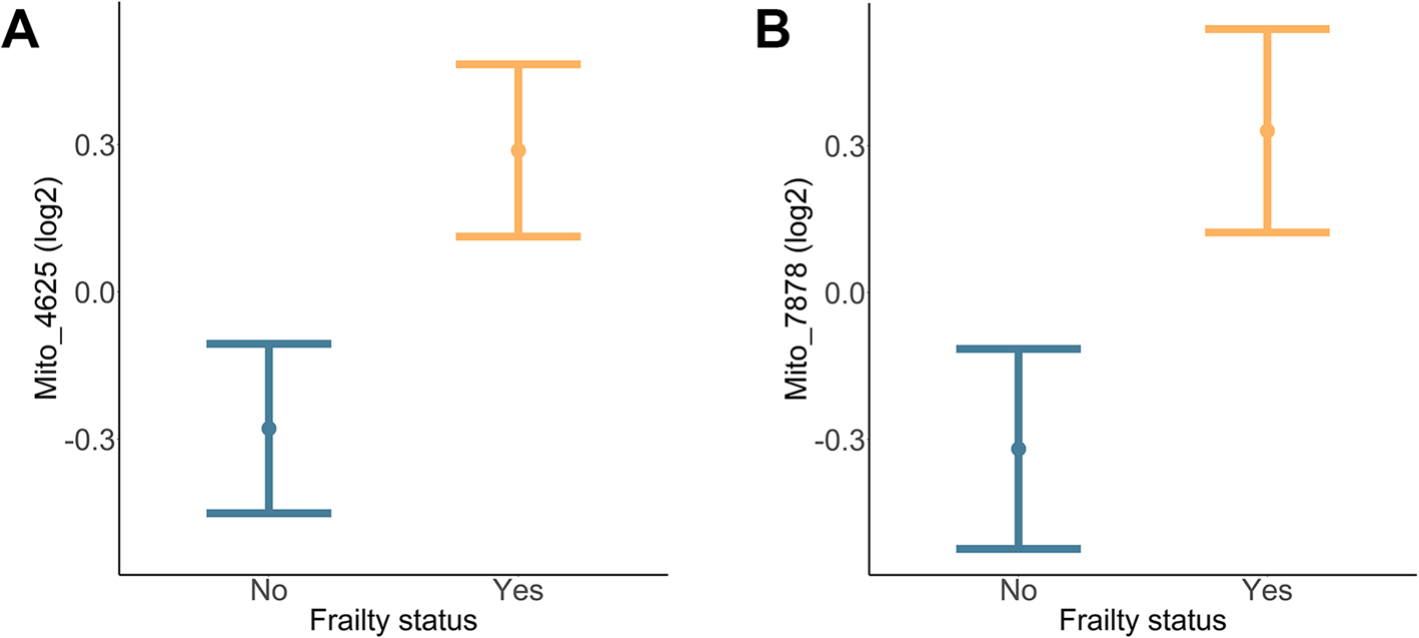
EV mtDNA levels are higher in frail individuals. Plasma EVs were isolated from participants in the frailty cohort (Table 1). DNA was then isolated from the EVs, and mtDNA levels were measured using mtDNA specific primers, targeting four regions of the mitochondrial genome (*n* = 177). EV mtDNA values were log_2_ transformed. Linear regression was used to examine the relationship between EV mtDNA levels and frailty status, accounting for sex, race, and poverty status. EV mtDNA levels amplified by the **A)** Mito_4625 primer sets and **B)** Mito_7878 primer sets were significantly higher in frail individuals. The plots show the linear regression values ± standard error of the estimated values

We used linear regression to analyze the relationship between EV mtDNA levels from each of the different primer sets with frailty status, sex, race, and poverty status. We found a significant frailty effect on EV mtDNA levels. EV mtDNA levels amplified with Mito_4625 (*p* = 0.023) and Mito_7878 (*p* = 0.027) were significantly higher in frail individuals compared to non-frail individuals (Fig. 2A, B). Therefore, frail individuals have higher EV mtDNA levels.

### Plasma EV inflammatory protein cargo is altered with frailty, race, sex, and poverty status

To further test whether other EV cargo was associated with frailty status, we quantified inflammatory proteins in EVs. Previously, we have found that EV inflammatory proteins are altered with mortality and diabetes mellitus status [36, 37]. We have also shown that inflammatory gene expression is altered with frailty status in a middle-aged, diverse cohort [42, 43]. To assess whether inflammatory protein content in EVs differed by frailty status, EVs were lysed and analyzed using a Multiplex Proximity Extension Assay (PEA). This quantitative and sensitive method is highly suitable to detect proteins in body fluids and in EVs [36, 37, 44–46]. In total, the panel analyzed the levels of 92 inflammatory proteins. Out of this pool, 14 proteins met our stringent threshold and were included in further analysis. These proteins are listed, along with their general functions in Supplementary Table 2. Using linear regression, several significant interactions of EV inflammatory proteins with frailty, race, sex, and poverty status were observed. These interactions are summarized in Supplementary Table 3 and detailed below. There were 10 different proteins that were significant in at least one of our analyses. There were four proteins that met our detection threshold but did not have significant interactions with frailty, race, sex, or poverty status: CD40, CXCL5, MMP-1, and VEGFA.

**Fig. 3:**
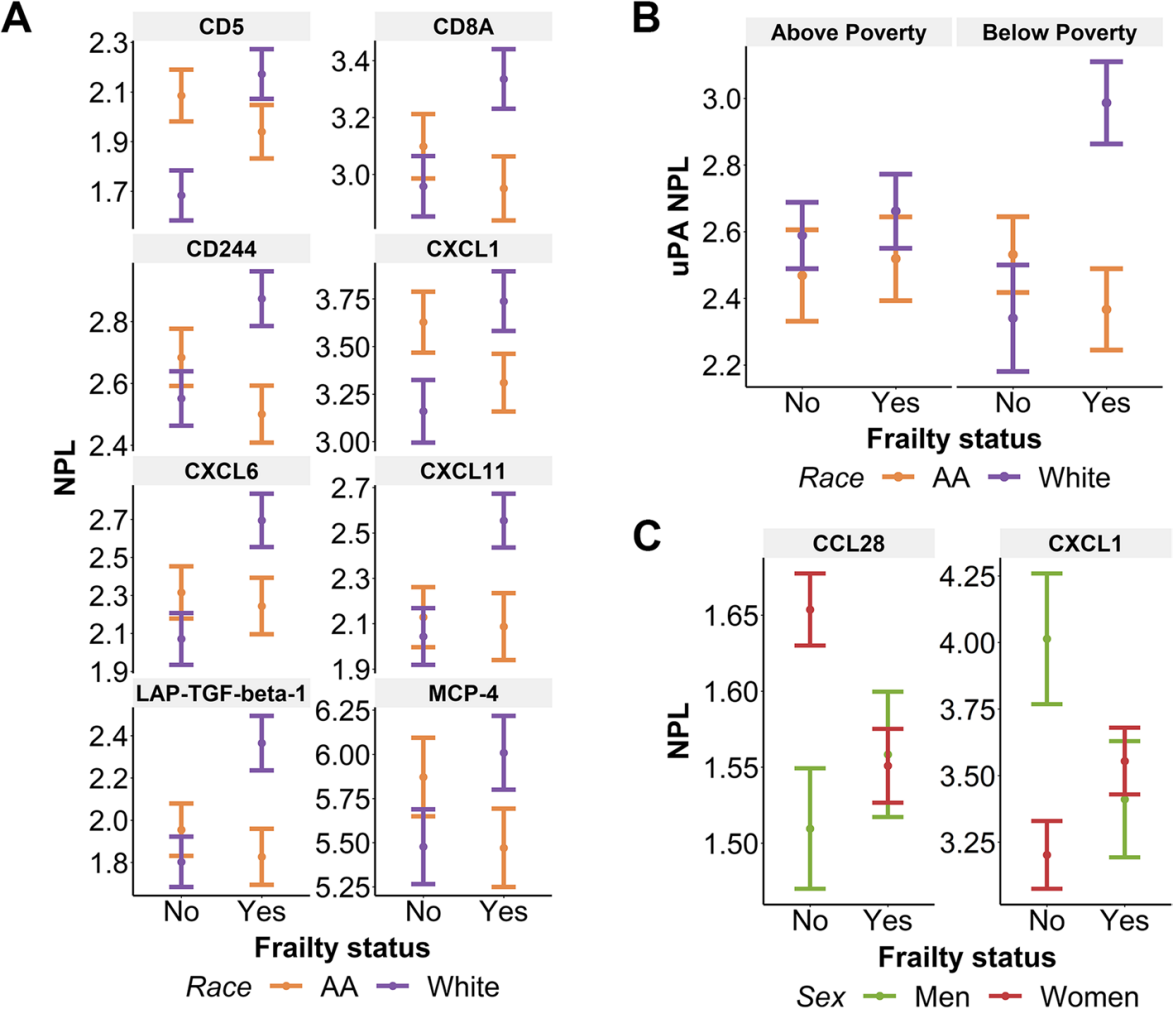
EV inflammatory proteins are associated with frailty, sex, and race. Plasma EVs from individuals in the frailty cohort were lysed and analyzed in a multiplex proximity extension assay (n = 177). Normalized protein levels (NPL, which is in a log_2_ scale) are shown. Linear regression was used to determine the relationship between EV protein levels and frailty status, accounting for sex, race, and poverty status. **A** EV inflammatory proteins that had a significant relationship between frailty status and race are plotted. The plots show the linear regression values ± standard error of the estimated values. **B** uPA had a significant three-way interaction with frailty status, race, and poverty status. **C** CCL28 and CXCL1 had a significant interaction with frailty and sex. AA = African American; NPL = normalized protein level

The EV levels of eight different proteins had significant interactions with frailty and race: T-cell surface glycoprotein CD5 (CD5) (*p* = 0.002), T-cell surface glycoprotein CD8 alpha chain (CD8A) (*p* = 0.017), natural killer cell receptor 2B4 (CD244; also known as SLAMF4) (*p* = 0.006), C-X-C motif chemokine ligand 1 (CXCL1) (p = 0.006), C-X-C motif chemokine ligand 6 (CXCL6) (*p* = 0.015), C-X-C motif chemokine ligand 11 (CXCL11) (*p* = 0.038), latency-associated peptide transforming growth factor beta-1 (LAP-TGF-beta-1) (*p* = 0.007), and monocyte chemotactic protein 4 (MCP-4; also known as CCL23) (*p* = 0.032). In general, inflammatory proteins were higher in frail White participants compared to both non-frail White and frail African American participants (Fig. 3A). Specifically, the EV levels of five proteins were lower in frail African American participants compared to frail White participants, CD8A, CD244, CXCL6, CXCL11, and LAP-TGF-beta-1 (Fig. 3A). Two proteins, CD5 and CXCL1, were higher in non-frail African American participants compared to non-frail White participants (Fig. 3A). Six of these proteins were higher in frail White participants compared to non-frail White participants, CD5, CD8A, CD244, CXCL6, CXCL11, and LAP-TGF-beta-1 (Fig. 3A). One protein, CXCL1, was lower in frail African American participants compared to non-frail African American participants (Fig. 3A). Thus, there were eight different EV-associated proteins that varied with frailty and race, and overall were highest in frail White participants.

The levels of one protein, urokinase-type plasminogen activator (uPA), had a significant interaction with frailty, race, and poverty status (*p* = 0.027). Here, there were differences between groups across poverty status by frailty and race. Frail White participants living below poverty had higher levels of uPA compared to frail White participants living above poverty (Fig. 3B). Amongst participants living below poverty, frail White participants had higher levels of uPA than both non-frail White participants and frail African American participants (Fig. 3B). Therefore, overall EV-associated uPA levels were highest in frail White participants living below poverty.

Two proteins had a significant interaction between frailty and sex, C-C motif chemokine ligand 28 (CCL28) (*p* = 0.023) and C-X-C motif chemokine ligand 1 (CXCL1) (*p* = 0.006). CCL28 was lower in frail women compared to non-frail women, and lower in non-frail men compared to non-frail women (Fig. 3C). Inversely, CXCL1 was higher in frail women compared to non-frail women, and higher in non-frail men compared to non-frail women (Fig. 3C). Additionally, CXCL1 levels were lower in frail men compared to non-frail men (Fig. 3C). Thus, CCL28 and CXCL1 levels were different with sex and frailty.

**Fig. 4:**
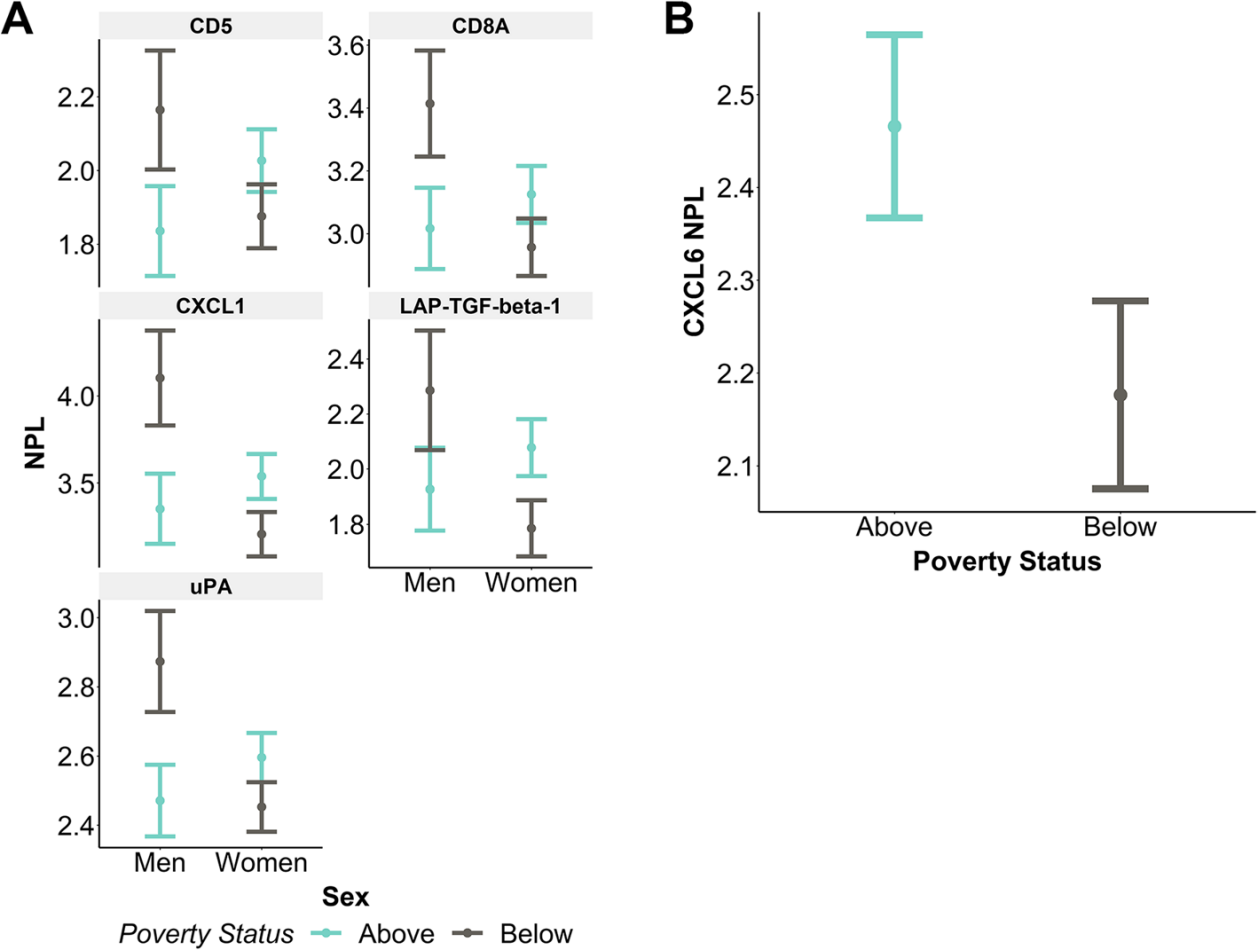
EV inflammatory proteins are associated with sex and poverty status. Plasma EVs from individuals in the frailty cohort were lysed and analyzed in a multiplex proximity extension assay (*n* = 177). Normalized protein levels (NPL, which is in a log_2_ scale) are shown. Linear regression was used to determine the relationship between EV protein levels and frailty status, accounting for sex, race, and poverty status (*n* = 177). **A** EV inflammatory proteins that had a significant association with sex and poverty status are plotted. The plots show the linear regression values ± standard error of the estimated values. **B** CXCL6 levels were significantly different with poverty status

Five proteins had significant interactions between sex and poverty status, CD5 (*p* = 0.042), CD8A (*p* = 0.023), CXCL1 (*p* = 0.006), LAP-TGF-beta-1 (*p* = 0.032), and uPA (*p* = 0.008). In general, all five proteins were highest in men living below poverty. Specifically, four of these proteins had higher levels in men living below poverty compared to women living below poverty, including CD8A, CXCL1, LAP-TGF-beta-1, and uPA (Fig. 4A). Two proteins, CXCL1 and uPA, were higher in men living below poverty compared to men living above poverty (Fig. 4A). Additionally, one protein, LAP-TGF-beta-1, was lower in women living below poverty compared to women living above poverty (Fig. 4A). CXCL6 had a significant association with poverty status (*p* = 0.045). CXCL6 was lower in individuals living below poverty compared to individuals living above poverty (Fig. 4B). Overall men living below poverty had the highest levels of five EV-associated proteins and one protein, CXCL6, was lower in participants living below poverty.

### Plasma EV mtDNA levels are significantly correlated with EV inflammatory proteins

**Fig. 5:**
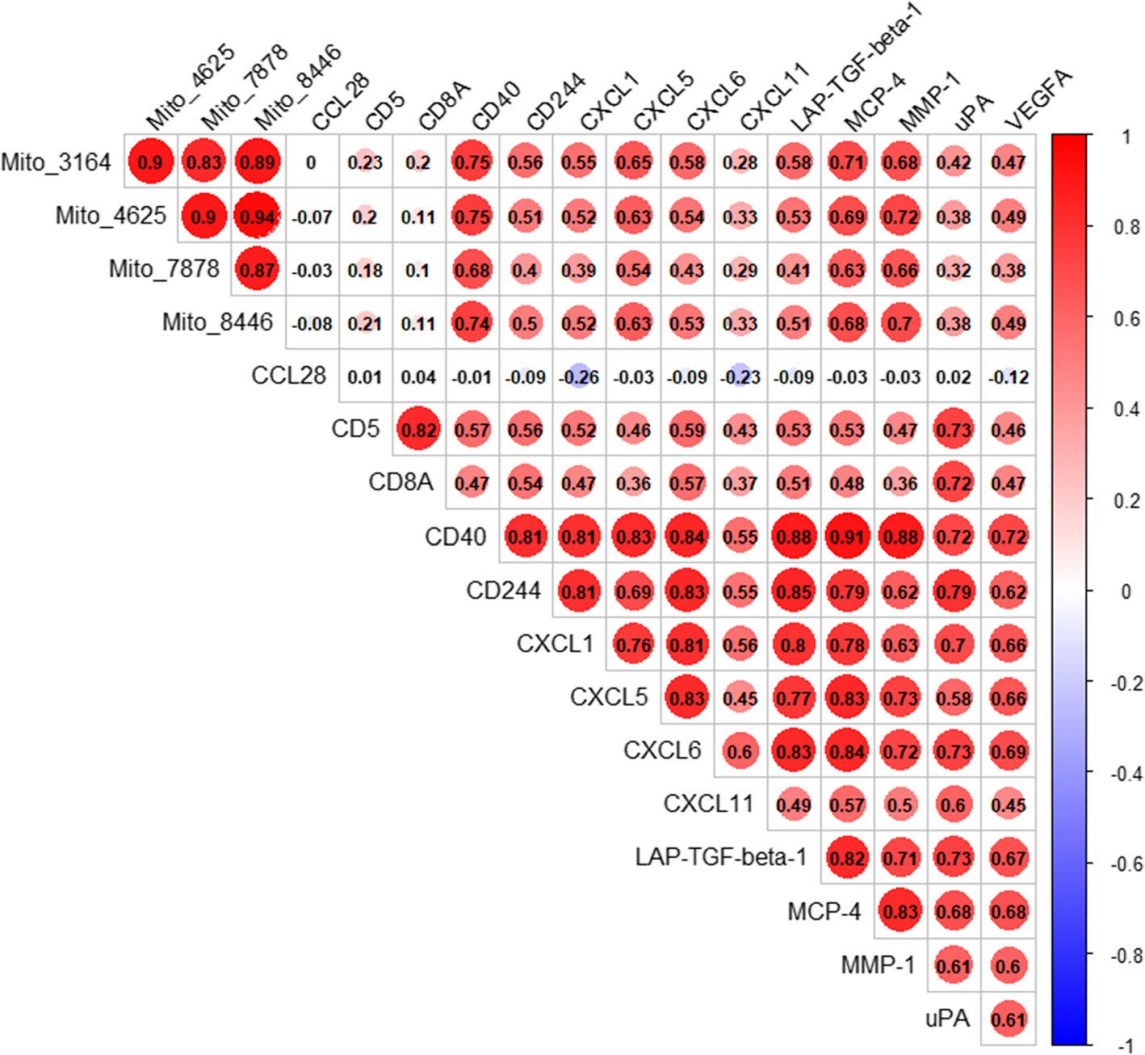
EV mtDNA levels are correlated with EV inflammatory proteins. Pearson correlation using pairwise complete observation was used to determine the associations between the four EV mtDNA amplicon regions and EV inflammatory proteins. Color is based on correlation coefficient (r), where r = 1 is red and r = − 1 is blue. Significance of the *p*-value is shown by size of the circle for each correlation. A p-value of 0.05 was used as the threshold for significance. Degree of significance is denoted by circle size. The four mitochondrial primer sets are denoted by “Mito_X”

To assess the relationships between EV levels of mtDNA and EV inflammatory proteins, we analyzed the correlation between the levels of these molecules using Pearson correlation. We included in our analysis all detectable inflammatory proteins in the EVs (Supplementary Table 2). We found that 12 of the 14 EV detectable inflammatory proteins were significantly positively correlated with all four EV mtDNA amplicon regions. (Fig. 5). One protein, CCL28 was not significantly correlated with EV mtDNA levels, but was negatively correlated with CXCL1 and CXCL11. All other inflammatory proteins significantly correlated to each other.

### EV inflammatory proteins and frailty

**Fig. 6:**
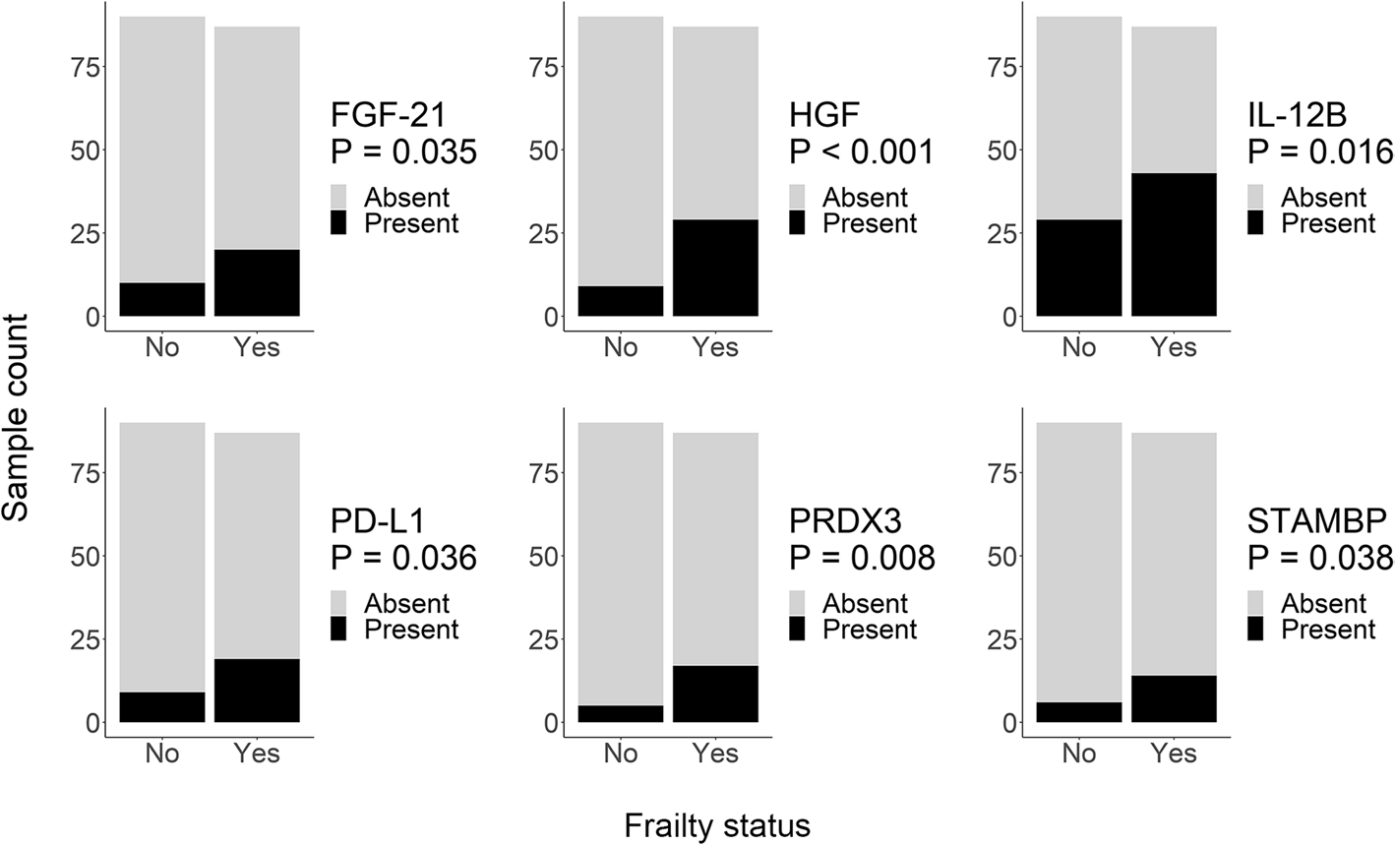
Presence of EV inflammatory proteins and frailty status. Plasma EVs were isolated from the frailty cohort, lysed, and analyzed using a multiplex proximity extension assay (*n* = 177). Logistic regression was used to assess the presence of each inflammatory protein in EVs with frailty status. The model also accounted for sex, race, and poverty status

Next, we wanted to determine whether the presence or absence, rather than level of inflammatory proteins, were associated with frailty status. We used logistic regression to examine the detection status of all 92 inflammatory proteins by frailty status, controlling for sex, race, and poverty status. The presence of six proteins were significantly associated with frailty status: fibroblast growth factor 21 (FGF-21), hepatocyte growth factor (HGF), interleukin-12 subunit beta (IL-12B), programmed death-ligand 1 (PD-L1), thioredoxin-dependent peroxide reductase, mitochondrial (PRDX3), and STAM-binding protein (STAMBP; also known as AMSH). EVs from frail individuals were more likely to contain these six proteins compared to EVs from non-frail individuals (Fig. 6).

## Discussion

We examined EVs in the context of frailty by performing a large-scale study of EVs from frail and non-frail middle-aged individuals in the context of race, sex, and poverty. EV concentration was higher in frail White individuals, and the DAMP, ccf-mtDNA, was higher in EVs from frail individuals. Inflammatory proteins were also more often present in EVs from frail individuals (FGF-21, HGF, IL-12B, PD-L1, PRDX3, and STAMBP). Notably, sex and the social determinants of health race and poverty status influenced the presence of inflammatory proteins in both African American and White adults.

Our findings with EV concentration are similar to another study that found higher levels of EV protein abundance, as a proxy for EV levels, in elderly frail (mean age = 78 years; *n* = 11) compared to non-frail (mean age = 74 years; *n* = 10) individuals in the BIOmarkers associated with Sarcopenia and Physical frailty in EldeRly pErsons (BIOSPHERE) cohort [35]. It should be noted that in this study frail individuals were classified as having sarcopenia as well. Immunoblotting was used to assess the levels of EV markers and mitochondrial proteins in these EVs. Using this semi-quantitative method, the authors reported lower levels of the mitochondrial proteins ATP5A, NDUFS3, and SDHB in participants with frailty and sarcopenia [35]. Another study found no differences in EV concentration in a small cohort of elderly (79–92 years) non-frail (*n* = 7) and frail (*n* = 5) individuals from Spain [33].

Mitochondrial components as EV cargo are an emerging topic of interest [21, 47]. Previously, we reported that EV mtDNA levels decline with advancing age [20], but are not associated with mortality [36]. Here we found that EV mtDNA levels were higher in frail participants versus non-frail participants. Although the four different primer sets were all significantly correlated with each other, only two primer sets against the NADH dehydrogenase 2 (*MT-ND2*) gene region (Mito_4625) and Cytochrome c oxidase subunit 2 (*COX2*) gene region (Mito_7878) were significantly associated with frailty. It is not known whether these differences are due to possible fragmentation of the mitochondrial genome, resistance of some gene regions to degradation, or variability in other regions. Nevertheless, these gene regions are adjacent on the mitochondrial genome, and only separated by the *COX1* gene, leading to the idea that there may be a biological explanation for the association of these mtDNA regions with frailty. Consistent with our data in EVs, whole plasma levels of ccf-mtDNA have been reported to be higher in frail individuals with late-life depression (> 65 years) [19]. In addition, ccf-mtDNA copy number was associated with frailty in individuals > 90 years [18].

Ccf-mtDNA can act as a DAMP and elicit a sterile immune response triggering inflammation [15]. Here we report that EV-associated inflammatory proteins are highly correlated with EV mtDNA levels. These data support the hypothesis that higher ccf-mtDNA in frail individuals may contribute to chronic systemic inflammation that may increase the vulnerability of frail individuals to environmental and endogenous stressors and may promote or accelerate the development of age associated disease.

Although we assayed 92 proteins only 14 met our stringent criteria for further analysis. Of these proteins, we found that protein levels had varying relationships with frailty and race (Fig. 3A), frailty, poverty, and race (Fig. 3B), frailty and sex (Fig. 3C), sex and poverty status (Fig. 4A), and poverty status (Fig. 4B). These complex relationships suggest that various factors can influence the inflammatory protein levels in EVs and that these factors should be considered when examining EV cargo [30]. For additional clarity we have compiled our data into a Table (Supplementary Table 3) and cross referenced with previous studies analyzing EV inflammatory protein levels in HANDLS sub-cohorts in the context of diabetes mellitus and mortality [36, 37]. Twelve proteins (8 in linear regression; 4 in presence/absence analysis) in our analysis were also significant in our various comparisons of individuals with or without diabetes or measures of disease severity [37]. As the FRAIL scale includes information about comorbidities, including diabetes mellitus, this may also indicate that diabetes mellitus may contribute to altered inflammatory protein content in EVs in frail individuals. Comparing to individuals with early mortality, only one protein, STAMBP, was significantly associated with frailty and mortality [36]. There is little known about this intracellular protein as EV cargo. However, it does play a role in regulating intracellular trafficking by deubiquitinating the endosomal sorting complexes required for transport (ESCRT) proteins. Future work lies in elucidating the biological mechanisms that contribute to STAMBP in plasma EVs.

There were higher levels of CD5, CD8A, CD244, CXCL1, CXCL6, CXCL11, LAP-TGF-beta-1, and MCP-4 in frail White individuals compared to frail/non-frail African American and non-frail White participants (Fig. 3A). Previously we reported genome-wide transcriptome changes in peripheral blood mononuclear cells with frailty and that these differences were race-dependent [43]. Biological pathways related to inflammatory and immune responses were differentially altered with frailty in African American and White adults. Like our data, CXCL1 was also higher in White individuals compared to African American individuals. Frailty prevalence in middle-age also varies by race. For example, frailty prevalence is higher in White adults aged 45–55 years compared to African American adults [3]. However, frailty prevalence is higher in African American adults compared to White adults when examined at older ages in the Cardiovascular Health Study (65–74 years) [48, 49] and in the Women’s Health and Aging Studies (70–79 years) [5]. Although differences in frailty prevalence may occur over the lifespan, these data point to race as an important SDOH that may differentially influence frailty.

Men living below poverty have higher levels of EV-associated CD5, CD8A, CXCL1, LAP-TGF-beta-1, and uPA compared to men living above poverty and women living below or above poverty. These data are intriguing given that living in poverty can be a lifelong stressor that can lead to “weathering” and the accelerated aging phenotype [50]. Living in poverty can have cumulative effects over the lifespan leading to adverse health outcomes, health disparities, and shortened lifespan. This chronic environmental stressor can drive biological transduction pathways that can affect transcriptional changes, inflammation, immune response as well as other pathways, for review [51]. In line with this idea, African American men living below poverty are particularly vulnerable to early mortality in the HANDLS study [52]. In a large meta-analysis of 1.7 million people, low socioeconomic status was a major risk factor for premature mortality [53]. Therefore, our finding that men living below poverty have higher levels of EV-associated inflammatory proteins provides a clue for how adversity can manifest resulting in heightened inflammation. This is especially important since inflammation drives many age-associated diseases.

In addition to examining levels of EV-associated inflammatory proteins, we also analyzed whether the presence of inflammatory proteins were different between frail and non-frail participants. We found that FGF-21, HGF, IL-12B, PD-L1, PRDX3, and STAMBP were more likely to be present in EVs from frail individuals compared to non-frail individuals. Interestingly, FGF-21 is a pleiotropic factor that plays complex roles in normal physiology and in pathological conditions. Elevated serum FGF-21 is associated with metabolic disorders, such as obesity and diabetes mellitus, as well as with mitochondrial diseases [54]. Plasma/serum FGF-21 has also been proposed as a potential biomarker of frailty [11]. However, current data are limited, and this hypothesis should be investigated further. PD-L1 is a ligand for the PD-1 immune checkpoint regulator and is an important mediator of immune escape of cancer cells [55]. Importantly, PD-L1 on circulating exosomes can be detected in healthy donors but the levels are significantly higher in patients with metastatic melanoma [56]. Exosomal PD-L1 may be important for the response and affect clinical outcomes to anti-PD-1 therapies [56]. Thus far, we do not fully understand the consequences of the presence of cancer-associated proteins in EVs and if the presence reflects normal physiology or a pathological process.

Our study has several limitations. Here we categorized frailty using the FRAIL scale, which was developed for utilization in community-based clinics [57, 58]. This measure is a broad construct that considers categories of physical fitness and health. Nevertheless, this construct has shown validity across comparative studies with other frailty measures for prediction of adverse outcomes and mortality [58, 59]. EV isolation remains a challenge in the field. Here we have used SEC to isolate EVs from plasma, which effectively removes soluble plasma proteins. The technological advancement of using the AFC for SEC for EV isolation allowed us to process samples in a high throughput manner and to date this is one of the largest scale studies using SEC. With this technique, we cannot exclude that there may be non-vesicular material that may co-precipitate during the isolation process. To circumvent this issue, we have implemented strict criteria for including proteins in our analysis and have included a DNase treatment step in our DNA isolation procedure to remove any DNA on the outside of the EVs. Our study is exploratory in nature warranting follow-up and validation in future studies.

## Conclusion

In our exploratory study, we report that EVs from frail middle-aged individuals had higher levels of the DAMP ccf-mtDNA, and that EV concentration was highest in frail White participants. Furthermore, EV-associated inflammatory proteins are altered with frailty, sex, race, and poverty status. EV inflammatory proteins are significantly correlated with EV mtDNA levels. Frail individuals are more likely to have several inflammatory proteins in their EVs versus non-frail individuals. These data suggest that EVs may carry DAMPs, such as mtDNA, as well as inflammatory proteins in frail individuals. This study provides clues to molecular mechanisms that may underlie frailty and point to possible avenues for guiding biomarker development or therapeutic intervention for frailty.

## Methods

### Clinical study participants

The study cohort was selected from the Healthy Aging in Neighborhoods of Diversity across the Life Span (HANDLS) study performed by the National Institute on Aging (NIA) Intramural Research Program (IRP), National Institutes of Health (NIH) [60]. HANDLS has been approved by the Institutional Review Board of the NIH and all participants provided written informed consent. HANDLS is a longitudinal, epidemiologic study comprised of community-dwelling adults in Baltimore, Maryland. The study is focused on examining the interaction of social, biological, and environmental factors on health disparities in aging and in the development and progression of age-associated illnesses. In this study, race was self-reported as either African American or White. Participants’ poverty status (above or below poverty) was based on household income at enrollment as defined by 125% of the 2004 U.S. Health and Human Services Poverty Guidelines [61].

For this study, we selected frail participants with available fasting blood samples between the ages of 45–55 years and randomly selected non-frail controls matched on race and sex. Due to sample availability, this final sub-cohort consisted of 177 participants (87 frail, 90 non-frail). Participants had a physical exam and were free from human immunodeficiency virus infection. The cohort information is listed in Table 1. Frailty was determined using a modified FRAIL scale, as previously reported [3]. Briefly, the FRAIL scale includes five categories, including fatigue, resistance (ability to climb stairs), ambulation (ability to walk a certain distance), number of illnesses, and loss of weight. Illness was assessed to be positive if participants reported a physician’s diagnosis for five or more of the following conditions: hypertension, diabetes, cancer, chronic lung disease, heart attack, congestive heart failure, angina, asthma, arthritis, stroke, and kidney disease. FRAIL scores are the number of components present and range from 0 (all components absent) to 5 (all components present). Participants were required to have data on at least three of the five components to be included in the sample, similar to the criteria used for other frailty studies [3, 48]. FRAIL scores are generally categorized into three frailty groups: frail (frail score 3–5), pre-frail (1-2), or non-frail (0) [57]. This study only included those either frail or non-frail.

Blood samples were collected in the morning after overnight fasting into sodium heparin collection tubes. For plasma isolation, Histopaque®-1077 (Sigma Aldrich, Cat: 10771) was slowly added to blood samples in 15 ml conical tubes and centrifuged for 20 min at 610 *g* with a slow deceleration. Upon successful separation, the top plasma layer was aliquoted and stored at − 80 °C.

### SEC EV isolation

Plasma (0.5 ml) samples were fractionated through size exclusion chromatography (SEC) with the AFC (IZON, Cat: AFC-V1) fitted with a qEVoriginal 70 nm column (IZON; Cat: SP1). Isolation specifications were followed using the default Collection Schedule for the qEV column. Briefly, the count/number of fractions was 10, size of fractions was 0.5 ml, and the buffer volume was left at the default collection volume. The columns were flushed with 15 ml 0.2 μm filtered phosphate buffered saline (PBS) prior to loading of the samples. The sample was loaded and run with 10 ml PBS. All 10 fractions were collected and the eluate from F1–3 were pooled together (1.5 ml), and collectively referred to as the EV-enriched fractions. F4–10 were kept separate. Additionally, 500 μl of the void volume was also collected separately. Columns were flushed with 15 ml PBS and were reused 5 times, according to the manufacturer’s recommendations. Samples were stored at − 80 °C for long-term storage.

### Quantification of protein concentration

EVs were isolated as described above from non-frail (*n* = 3) and frail (*n* = 3) individuals. All fractions were lysed in a 10X lysis buffer (10X Tris-buffered saline (TBS), 10% TritonX-100, 20 mM Ethylenediaminetetraacetic acid (EDTA) with protease and phosphatase inhibitors). Final concentration of the lysis buffer was 1X. Protein concentration was calculated using the Bradford Assay using a standard curve of bovine serum albumin (BSA). Samples were run in duplicate and absorbance at 595 nm was read on a SpectraMax M2 Microplate Reader (Molecular Devices, LLC). For each sample, the fractions were normalized to the mean of the EV fractions (F1–3), respective of frailty status.

### Immunoblotting

EV-enriched fractions (F1–3), as well as F4–10 and the void volume were lysed (1:10) in a 10X lysis buffer as described above. SEC fractions (~ 7 μg), and an equal volume of the void fraction were loaded compared to the EV-enriched fraction. Human umbilical vein endothelial cell (HUVEC) lysate was used as a positive control and thus more protein (~ 37 μg) was loaded to ensure that EV markers were visible. Samples were run on 4–12% NuPAGE Bis-Tris gel under sodium dodecyl sulfate (SDS)-denaturing conditions (Invitrogen ThermoFisher Scientific) and transferred onto polyvinylidene difluoride (PVDF). After blocking in 3% BSA in TBS with 0.1% Tween® 20 Detergent, the membrane was incubated with primary antibodies for 1 h at room temperature: CD9 (System Biosciences EXOAB-CD9A-1), CD81 (System Biosciences EXOAB-CD81A-1), Flotillin-1 (Abcam ab133497), GM130 (Abcam ab52649), and ApoA1 (Abcam ab64308). All primary antibodies were diluted 1:500. For detection, the membranes were incubated with the appropriate secondary horseradish peroxidase (HRP)-conjugated antibodies at 1:5000 dilutions for 32 min. These blots were visualized with the KwikQuant Ultra HRP Substrate Solution and imaging system according to the manufacturer’s protocol (Kindle Biosciences, LLC; Cat: R1002).

### Exo-check™ exosome antibody array

We analyzed EV markers using Exo-Check**™** Exosome Antibody Array (System Biosciences; Cat #: EXORAY200A-4). SEC-isolated EV-enriched fractions (F1–3) were lysed following the manufacturer’s procedure. The blot was visualized with the KwikQuant Ultra HRP Substrate Solution and imaging system were used according to the manufacturer’s protocol (Kindle Biosciences, LLC; Cat: R1002).

### Nanoparticle tracking analysis

SEC-isolated EVs were diluted into 0.2 μm filtered PBS. Different dilutions were used due to variation in concentration and the dilution factor was adjusted for when calculating the concentration per sample. Size distribution and concentration were analyzed using nanoparticle tracking analysis (NTA) on a NanoSight NS500 (Malvern Panalytical, software version NTA 3.4 Build 3.4.4). Samples were recorded in five videos of 20 sec at camera level 16 and detection level 4. Samples were analyzed on the same machine by one user. Total EV concentration from plasma was calculated as previously described [62]. EV concentration values were log_2_ transformed as they were positively skewed.

### Electron microscopy

Electron microscopy was performed by the Johns Hopkins University School of Medicine Microscope Facility. SEC-isolated EVs were absorbed to freshly ionized 400 mesh formvar/carbon coated grids (Electron Microscopy Sciences, Cat: CF400-Cu-UL) and then washed with TBS (3 drops) and negatively stained in 1% aqueous uranyl acetate. Images were then taken with a transmission electron microscope (ThermoFisher Talos L120C) at 120 kV using a ThermoFisher Ceta 16mP 16bit CMOS camera. Original image was zoomed in retaining all information including scale using Adobe Photoshop.

### EV DNA isolation

Previously, we established an experimental pipeline for analyzing EV mtDNA levels [20]. Here we have optimized this protocol for use of SEC-isolated EVs and details of the experimental workflow are in Supplementary Fig. 1. For DNA isolation from the SEC EVs, 155 μl of each sample was DNase treated (Lucigen, Cat: DB0715K; 5 U) to remove any DNA on the surface of the EVs at 37 °C for 30 min. The reaction was stopped by the addition of 20 μl DNase Stop Solution at 65 °C for 10 min. DNA was isolated following the DNeasy Blood and Tissue kit protocol (Qiagen, Cat: 69506). An additional spin was conducted at 20,000 *g* for 1 min after adding Buffer AW2, and new waste collection tubes were used in between each spin. Additionally, a 5 min incubation at room temperature of 50 μl AE Buffer in the spin column was added before the final 1 min 8000 *g* spin for DNA elution. The eluted DNA (~ 50 μl) was further diluted in an additional 50 μl of AE Buffer (Qiagen) and stored at − 20 °C.

### Quantitative real time-PCR

Quantitative real-time PCR (qPCR) analysis was performed as previously reported [20]. Briefly, each reaction was a total of 13 μl and included mitochondrial gene-specific primers (2.5 μl/rxn), TaqMan™ Fast Advanced Master Mix (7.5 μl/rxn), and DNA isolated from the EVs (3 μl/rxn). The primer design is described in [20], and primer sequences are listed in Supplemental Table 2. A 7900HT Fast Real-Time PCR System was used to run the samples (Applied Biosystems, software version SDS 2.4.1). The thermal profile used is as follows: 50 °C for 2 min, 95 °C for 10 min, followed by 40 cycles of 15 sec at 95 °C, and 1 min at 60 °C. mtDNA levels were calculated as previously reported [20]. EV mtDNA values were log_2_ transformed as they were positively skewed.

### Multiplex proximity extension assay

SEC-isolated EVs were lysed in 10X lysis solution (described above) with protease and phosphatase inhibitors. Protein concentration was calculated as above and processed similarly as previously described [37]. Here, 60 μg of protein in 40 μl (f.c 1.5 μg/μl) of each EV lysate were added to 96-well plates, and then analyzed with Olink® Proteomics biomarker Inflammation Panel using Proximity Extension Assay (PEA) technology (Olink® Proteomics). Experiments were performed blind of group status. Internal controls were used in each step, including a negative control that accounted for background levels and an inter-plate control that accounted for different plates. Protein data were normalized to inter- and intra-assay controls and represented as normalized protein expression (NPX) units on a log_2_ scale, referred to here as normalized protein level (NPL). In total, 92 proteins were tested using the Olink® Inflammation panel. Out of the 92 proteins, 14 proteins met our threshold for being less than or equal to 35% at the lower limit of detection, meaning that each of those proteins was present in more than 65% of all the EV samples. Any missing protein values were excluded from the analysis.

### Statistics

Statistical analysis was performed using R software (software version R 4.2.0) [63]. Student’s t-test was used to analyze differences between groups for age. Pearson’s chi-squared test was used to test for differences across sex, race, and poverty status. Correlations between EV mtDNA levels were assessed by Pearson correlation (pairwise complete observation). EV concentration, EV mtDNA levels, and EV inflammatory protein levels were analyzed using linear regression, which was modeled to the study design of frailty status, sex, race, and poverty status. Backward stepwise regression was used for all linear regression models starting with a full model considering all possible three-way interactions and statistical significance based on the relevant coefficient in the model. Non-significant interactions were eliminated until a final model, or base model of all main effects, was achieved. All models included frailty status, sex, race, and poverty status as main effects. Presence of EV proteins were analyzed using threshold of detection data via logistic regression, including the study design of frailty status, sex, race, and poverty status. Statistical significance was defined as a *p*-value < 0.05.

## Supplementary Information

**Additional file 1: Supplementary Information. Supplementary Table 1.** Primer sequences for mitochondrial DNA qPCR. **Supplementary Table 2.** Inflammatory proteins detected in plasma EVs. **Supplementary Table 3.** Significant interactions of EV inflammatory proteins. **Supplementary Figure 1.** Schematic workflow and primer design for quantifying EV mtDNA levels. **Supplementary Figure 2.** Positive correlation between EV mtDNA levels.

## Data Availability

The datasets generated and analyzed during the current study are available from the corresponding author on reasonable request through the HANDLS website https://handls.nih.gov/.

## Abbreviations

AFC: Automated Fraction Collector
AA: African American
BSA: Bovine serum albumin
ccf-DNA: circulating cell-free DNA
ccf-mtDNA: circulating cell-free mitochondrial DNA
DAMP: Damaged associated molecular pattern
EDTA: Ethylenediaminetetraacetic acid
EV: Extracellular vesicle
h: hour
HRP: Horseradish peroxidase
HUVEC: Human umbilical vein endothelial cell
IRP: Intramural Research Program
miRNA: microRNA
min: minute
mtDNA: mitochondrial DNA
NIA: National Institute on Aging
NIH: National Institutes of Health
NPX: Normalized protein expression
NPL: Normalized protein level
NTA: Nanoparticle tracking analysis
PBS: Phosphate buffered saline
PEA: Proximity Extension Assay
PVDF: Polyvinylidene difluoride
qPCR: quantitative real-time PCR
SD: Standard deviation
SDOH: Social determinants of health
SD: Standard deviation
SDS: Sodium dodecyl sulfate
sec: second
SEC: Size exclusion chromatography
TBS: Tris-buffered saline
