# Supplementary material for: Mitochondrial DNA and inflammatory proteins are higher in extracellular vesicles from frail individuals
Source: Immun Ageing. 2023 Jan 30;20:6. doi: 10.1186/s12979-023-00330-2 (PMC9885591; doi:10.1186/s12979-023-00330-2)
Supplement: Supplementary file 1 — Additional file 1: Supplementary Information. Supplementary Table 1. Primer sequences for mitochondrial DNA qPCR. Supplementary Table 2. Inflammatory proteins detected in plasma EVs. Supplementary Table 3. Significant interactions of EV inflammatory proteins. Supplementary Figure 1. Schematic workflow and primer design for quantifying EV mtDNA levels. Supplementary Figure 2. Positive correlation between EV mtDNA levels. [file 12979_2023_330_MOESM1_ESM.pdf]

**Additional File 1: Supplementary Information**

# Mitochondrial DNA and Inflammatory Proteins are Higher in Extracellular Vesicles from Frail Individuals

Anjali M. Byappanahalli, Nicole Noren Hooten, Mya Vannoy, Nicolle A. Mode, Ngozi Ezike, Alan B. Zonderman and Michele K. Evans

## Supplementary Table 1. Primer sequences for circulating cell-free mitochondrial DNA qPCR

| Gene | Primer Name | Forward Sequence / Reverse Sequence | Probe | Size (bp) | Ref. |
|---|---|---|---|---|---|
| MT-RNR2/MT-TL1 | Mito_3164 | 5'CCTTCCCCCGTAAATGATATCA3' / 5'GCCATCTTAACAAACCCTGTTCTT3' | 5'FAM-AACTTAGTATTATACCCACACCC-MGB3' | 76 | (1) |
| MT-ND2 | Mito_4625 | 5'CACAGAAGCTGCCATCAAGTA3' / 5'CCGGAGAGTATATTGTTGAAGAG3' | 5'FAM-CCTCACGCAAGCAACCGCATCC-BLACKHOLE-3' | 89 | (2) |
| MT-COX2 | Mito_7878 | 5'AATCAATTGGCGACCAATGG3' / 5'CGCCTGGTTCTAGGAATAATGG3' | 5'FAM-ACTGAACCTACGAGTACAC-MGB-3' | 100 | (3) |
| MT-ATP8 | Mito_8446 | 5'AATATTAAACACAAACTACCACCTACCT3' / 5'TGGTTCTCAGGGTTTGTTATAA3' | 5'-FAM-CCTCACCAAAGCCCATA-MGB-3' | 79 | (4) |

**Supplementary Table 2. Inflammatory proteins detected in plasma EVs**

| Protein Symbol | Protein Name | Protein function | Ref |
|---|---|---|---|
| CCL28 | C-C motif chemokine ligand 28 | chemokine; antimicrobial activity; immune responses | (5) |
| CD5 | T-cell surface glycoprotein CD5 | scavenger receptor; immunomodulatory function, pattern recognition receptors | (6) |
| CD8A | T-cell surface glycoprotein CD8 alpha chain | cell surface receptor on cytotoxic T cells; mediates immune cell-cell interactions | (7) |
| CD40 | Cluster of differentiation 40 | co-stimulatory receptor on antigen-presenting cells | (8) |
| CD244 | Natural killer cell receptor 2B4 | receptor involved in immune regulation; cytotoxicity; cytokine production | (9) |
| CXCL1 | C-X-C motif chemokine ligand 1 | chemokine; angiogenesis, inflammatory responses | (10) |
| CXCL5 | C-X-C motif chemokine ligand 5 | chemokine; promotes angiogenesis and tumorigenesis, remodels connective tissue | (11) |
| CXCL6 | C-X-C motif chemokine ligand 6 | chemokine; recruits neutrophils for anti-microbial actions | (12) |
| CXCL11 | C-X-C motif chemokine ligand 11 | proinflammatory chemokines; inflammation and response to infection | (13) |
| LAP-TGF-β-1 | Latency-assoc. peptide transforming growth factor β-1 | binds and maintains TGFβ latency | (14) |
| MCP-4 | Monocyte chemotactic protein 4 | chemokine; role in chronic inflammatory diseases | (15) |
| MMP-1 | Matrix metalloproteinase-1 | breaks down extracellular matrix proteins in physiological and pathological processes | (16) |
| uPA | Urokinase-type plasminogen activator | serine protease that converts inactive plasminogen to plasmin | (17) |
| VEGFA | Vascular endothelial growth factor A | mitogen that acts on endothelial cells and promotes vasculogenesis, angiogenesis | (18) |

Inflammatory proteins detected in EVs are listed. These 14 proteins met our threshold for being present in more than 65% of all the EV samples. General functions of proteins are listed, not necessarily functions attributed to these proteins in EVs. Note: MCP-4 is also known as CCL23 and CD244 is also known as SLAMF4. assoc. = associated

**Supplementary Table 3. Significant interactions of EV inflammatory proteins**

| Protein | Sex × Poverty | Frailty × Sex | Frailty × Race | Frailty × Race × Poverty |
|---|---|---|---|---|
| CCL28 | | * | | |
| CD5 | * | | ** | |
| CD8A | * | | * | |
| CD40 | | | | |
| CD244 | | | ** | |
| CXCL1 | ** | * | ** | |
| CXCL5 | | | | |
| CXCL6+ | | | * | |
| CXCL11 | | | * | |
| LAP-TGF-beta-1 | * | | ** | |
| uPA | ** | | | * |
| MCP-4 | | | * | |
| MMP-1 | | | | |
| VEGFA | | | | |

Significant interactions are indicated for the inflammatory proteins detected in EVs. These significant interactions were determined by linear regression analysis modeled to the study design of frailty, sex, race, and poverty status. *P<0.05; **P<0.01
+ CXCL6 also had a significant poverty status effect.

## Supplementary Figure 1

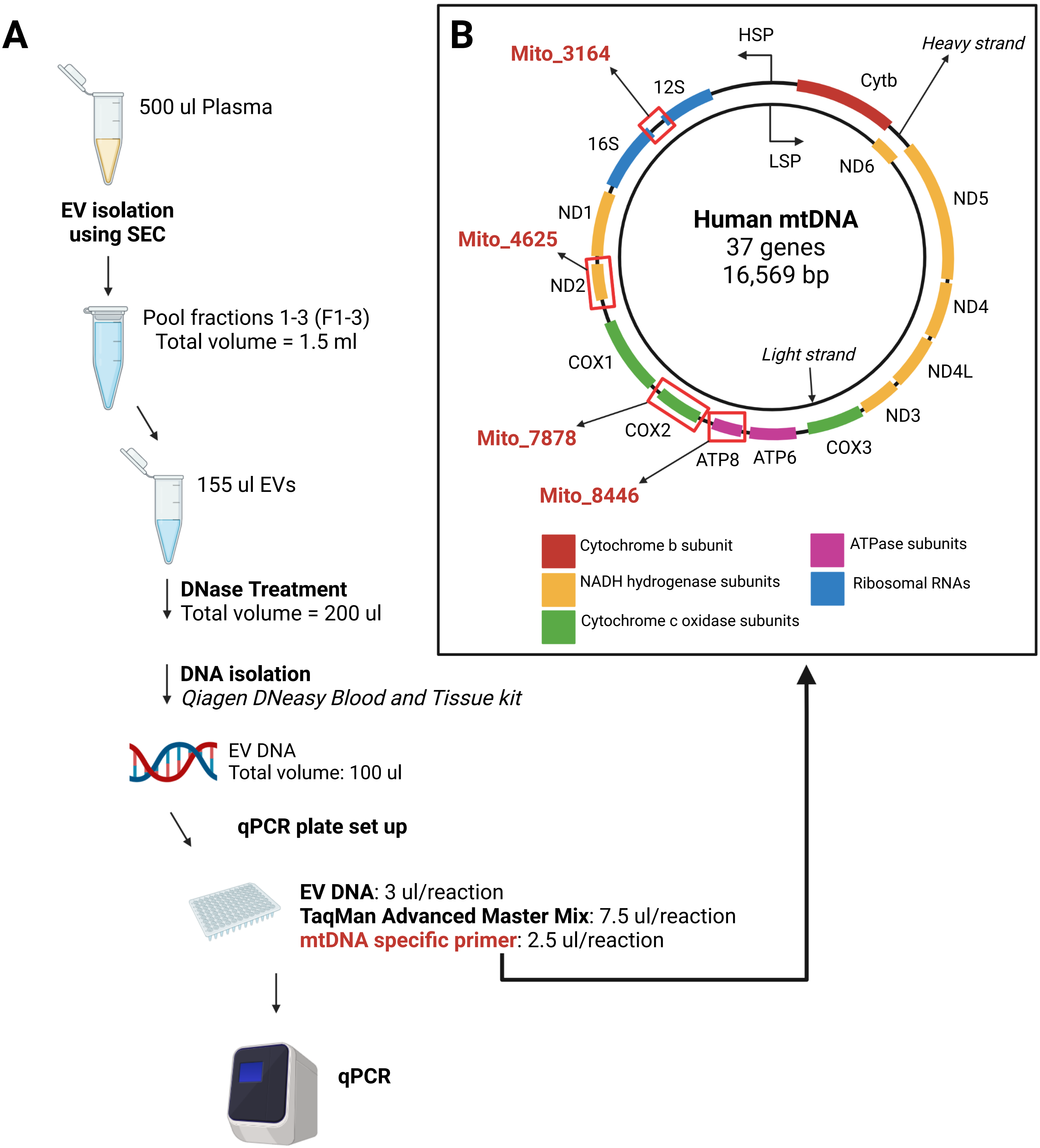


**Supplementary Figure 1. Schematic workflow and primer design for quantifying EV mtDNA levels. (A)** Schematic of experimental workflow for quantifying mitochondrial DNA from size exclusion chromography (SEC) isolated extracellular vesicles (EVs). **(B)** Mitochondrial genome with mtDNA primer regions indicated by a red box and primer name denoted by starting nucleotide. qPCR=quantitative real-time PCR

## Supplementary Figure 2

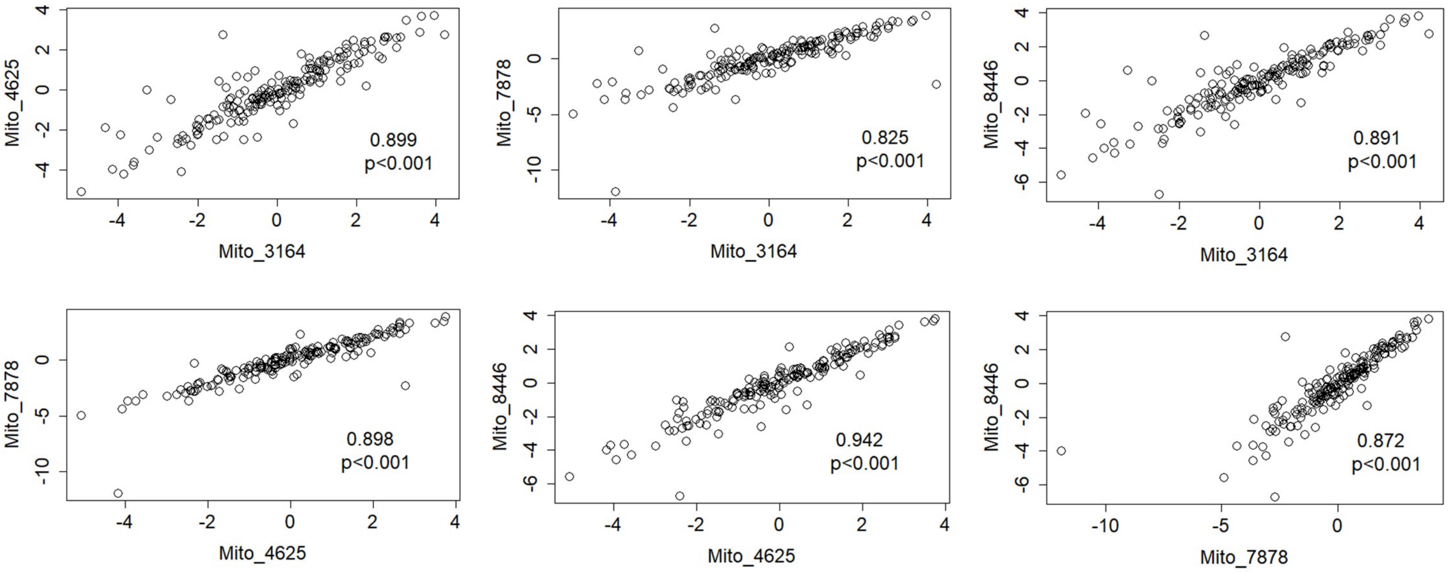


**Supplementary Figure 2. Positive correlation between EV mtDNA levels.** Plasma EVs were isolated from participants in the frailty cohort (Table 1). DNA was isolated from EVs and mtDNA levels were measured using mtDNA specific primers (Supplementary Table 1), targeting four regions of the mitochondrial genome. The relationship between EV mtDNA levels (log2 transformed) were analyzed by Pearson correlation. r values and p values are indicated.

## Supplementary Information References


1. Lazo S, Noren Hooten N, Green J, Eitan E, Mode NA, Liu Q-R, et al. Mitochondrial DNA in extracellular vesicles declines with age. Aging cell. 2021;20(1):e13283.
2. Pinti M, Cevenini E, Nasi M, De Biasi S, Salvioli S, Monti D, et al. Circulating mitochondrial DNA increases with age and is a familiar trait: Implications for "inflamm-aging". Eur J Immunol. 2014;44(5):1552-62.
3. Shao JY, Gao HY, Li YH, Zhang Y, Lu YY, Zeng YX. Quantitative detection of common deletion of mitochondrial DNA in hepatocellular carcinoma and hepatocellular nodular hyperplasia. World J Gastroenterol. 2004;10(11):1560-4.
4. Walker JA, Hedges DJ, Perodeau BP, Landry KE, Stoilova N, Laborde ME, et al. Multiplex polymerase chain reaction for simultaneous quantitation of human nuclear, mitochondrial, and male Y-chromosome DNA: application in human identification. Analytical Biochemistry. 2005;337(1):89-97.
5. Mohan T, Deng L, Wang B-Z. CCL28 chemokine: An anchoring point bridging innate and adaptive immunity. International Immunopharmacology. 2017;51:165-70.
6. Velasco-de Andrés M, Casadó-Llombart S, Català C, Leyton-Pereira A, Lozano F, Aranda F. Soluble CD5 and CD6: Lymphocytic Class I Scavenger Receptors as Immunotherapeutic Agents. Cells. 2020;9(12):2589.
7. Ellmeier W, Haust L, Tschismarov R. Transcriptional control of CD4 and CD8 coreceptor expression during T cell development. Cell Mol Life Sci. 2013;70(23):4537-53.
8. Díaz Á, González-Alayón I, Pérez-Torrado V, Suárez-Martins M. CD40-CD154: A perspective from type 2 immunity. Seminars in immunology. 2021;53:101528.
9. Sun L, Gang X, Li Z, Zhao X, Zhou T, Zhang S, et al. Advances in Understanding the Roles of CD244 (SLAMF4) in Immune Regulation and Associated Diseases. Front Immunol. 2021;12:648182.
10. Korbecki J, Barczak K, Gutowska I, Chlubek D, Baranowska-Bosiacka I. CXCL1: Gene, Promoter, Regulation of Expression, mRNA Stability, Regulation of Activity in the Intercellular Space. Int J Mol Sci. 2022;23(2).
11. Zhang W, Wang H, Sun M, Deng X, Wu X, Ma Y, et al. CXCL5/CXCR2 axis in tumor microenvironment as potential diagnostic biomarker and therapeutic target. Cancer Communications. 2020;40(2-3):69-80.
12. Rajarathnam K, Schnoor M, Richardson RM, Rajagopal S. How do chemokines navigate neutrophils to the target site: Dissecting the structural mechanisms and signaling pathways. Cellular signalling. 2019;54:69-80.
13. Metzemaekers M, Vanheule V, Janssens R, Struyf S, Proost P. Overview of the Mechanisms that May Contribute to the Non-Redundant Activities of Interferon-Inducible CXC Chemokine Receptor 3 Ligands. Front Immunol. 2018;8(1970).
14. Robertson IB, Horiguchi M, Zilberberg L, Dabovic B, Hadjiolova K, Rifkin DB. Latent TGF-β-binding proteins. Matrix Biol. 2015;47:44-53.
15. Mendez-Enriquez E, García-Zepeda EA. The multiple faces of CCL13 in immunity and inflammation. Inflammopharmacology. 2013;21(6):397-406.
16. Bassiouni W, Ali MAM, Schulz R. Multifunctional intracellular matrix metalloproteinases: implications in disease. The FEBS journal. 2021;288(24):7162-82.

17. Mahmood N, Mihalcioiu C, Rabbani SA. Multifaceted Role of the Urokinase-Type Plasminogen Activator (uPA) and Its Receptor (uPAR): Diagnostic, Prognostic, and Therapeutic Applications. Front Oncol. 2018;8:24.
18. Zhang Y, Brekken RA. Direct and indirect regulation of the tumor immune microenvironment by VEGF. Journal of Leukocyte Biology. 2022;111(6):1269-86.
